# The expression of one ankyrin *pk2* allele of the WO prophage is correlated with the *Wolbachia* feminizing effect in isopods

**DOI:** 10.1186/1471-2180-12-55

**Published:** 2012-04-12

**Authors:** Didier Bouchon, Chao Liu, Lanming Chen, Roger A Garrett, Pierre Grève

**Affiliations:** 1 Ecologie, Evolution, Symbiose, UMR CNRS 6556, Université de Poitiers, Poitiers, 86022, France; 2 Zoological Institute, University of Basel, Basel, 4051, Switzerland; 3 Department of Biology, University of Copenhagen, Copenhagen, 2200 N, Denmark

## Abstract

**Background:**

The maternally inherited α-Proteobacteria *Wolbachia pipientis* is an obligate endosymbiont of nematodes and arthropods, in which they induce a variety of reproductive alterations, including Cytoplasmic Incompatibility (CI) and feminization. The genome of the feminizing *w*VulC *Wolbachia* strain harboured by the isopod *Armadillidium vulgare* has been sequenced and is now at the final assembly step. It contains an unusually high number of ankyrin motif-containing genes, two of which are homologous to the phage-related *pk1* and *pk2* genes thought to contribute to the CI phenotype in *Culex pipiens*. These genes encode putative bacterial effectors mediating *Wolbachia*-host protein-protein interactions *via* their ankyrin motifs.

**Results:**

To test whether these *Wolbachia* homologs are potentially involved in altering terrestrial isopod reproduction, we determined the distribution and expression of both *pk1* and *pk2* genes in the 3 *Wolbachia* strains that induce CI and in 5 inducing feminization of their isopod hosts. Aside from the genes being highly conserved, we found a substantial copy number variation among strains, and that is linked to prophage diversity. Transcriptional analyses revealed expression of one *pk2* allele (*pk2b2*) only in the feminizing *Wolbachia* strains of isopods.

**Conclusions:**

These results reveal the need to investigate the functions of *Wolbachia* ankyrin gene products, in particular those of Pk2, and their host targets with respect to host sex manipulation.

## Background

The evolutionary success of the maternally inherited α-Proteobacteria *Wolbachia pipientis* is partly due to its ability to manipulate host reproduction to favour vertical transmission from mother to offspring. *Wolbachia* are also able to switch between hosts *via* horizontal transfer, which contributes to the impressive diversity and range of infected hosts [1]. These obligate endosymbionts are found in most filarial nematodes and are estimated to be present in ~60% of arthropod species [2-4]. In arthropods, *Wolbachia* are considered to be sex-parasites because they alter compatibility between eggs and sperm, feminize or kill males, or induce parthenogenesis [2,5,6]. Since *Wolbachia* remain unculturable endosymbionts, comparative genomics and evolutionary approaches are particularly useful for identifying putative bacterial determinants involved in *Wolbachia*-host interactions.

Recent genome analyses of different *Wolbachia* strains revealed a surprisingly high number of ankyrin domain-containing genes (*ank* genes) [7-11]. Their presence is suggested to be the result of lateral gene transfer since they are mostly found in eukaryotes but in few bacterial and viral genomes [12,13]. The 33-residue ankyrin repeats (ANK) form tandem arrays that mediate specific protein-protein interactions and have diverse functions in transcription initiation, cell cycle regulation and signalling, cytoskeleton integrity, ion transport, inflammatory responses and development [12,14]. The two closely related intracellular bacteria *Anaplasma phagocytophilum* and *Ehrlichia chaffeensis* secrete ankyrin proteins (AnkA and p200, respectively) that bind to host DNA and/or proteins [15,16]. It has been demonstrated that AnkA plays an important role in facilitating intracellular infection [17] whereas p200 is thought to affect host cell gene transcription and promote the survival of the pathogen [16]. Hence it has been suggested that *ank* genes encode *Wolbachia* effectors that alter host biology [18,19].

Several studies have suggested that *Wolbachia* ANK proteins were implicated in the molecular basis of Cytoplasmic Incompatibility (CI) [8,9,20-23]. Sequence divergence between closely related *Wolbachia* strains causing distinct CI types in *Culex pipiens* mosquito populations has been found in two *ank* genes (*pk1* and *pk2*), among a subset of phylogenetic markers [20,22-25]. Thus, these polymorphic *pk1* and *pk2 ank* genes, located within a so-called WO prophage region of *Wolbachia* genome, are suggested to contribute to the CI phenotype. Consistent with this argument, expression of the *pk2* gene occurred specifically in female mosquitoes [8,22,23]. Moreover, a premature stop codon was found in the *pk2* gene of the *Wolbachia* strain (*w*Au) that is unable to cause CI in *D. simulans*[21].

In this study, we aimed to determine whether the prophage *pk1* and *pk2* ankyrin genes were involved in the CI phenotype described in three *Wolbachia*-infected species of terrestrial isopods. We also investigated whether these genes were conserved and expressed in *Wolbachia* strains inducing feminization, the main *Wolbachia* phenotype described for this group of hosts [2]. From the genome of the feminizing *w*VulC *Wolbachia* strain that infects the isopod *Armadillidium vulgare* (the genome completion is currently being done by our group in the frame of the European *Wolbachia* project: EuWol), we annotated the *pk1* and *pk2* alleles among all *ank* genes identified from the *w*VulC contigs. We investigated the distribution, copy number and expression patterns of both genes in seven additional *Wolbachia* strains that induce either CI or feminization in isopods. We identified a large copy number variation of the *pk1* and *pk2* genes among *Wolbachia* strains, which is probably coupled to prophage evolution. Surprisingly, our results also revealed that expression of one *pk2* allele (*pk2b2*) is only detected in feminizing *Wolbachia* strains and never in the three CI-inducing strains of isopods.

## Results

### Characterization and distribution of *pk1* and *pk2* genes

Six copies of the *pk1* gene and three copies of the *pk2* gene were identified in the contig assembly of the *w*VulC genome (Table 1). Each of the six putative prophage regions of the assembly contains one *pk1* allele and three of these prophages also harbour one *pk2* allele (Table 1). Two *w*VulC *pk1* alleles (ANK46a/b and ANK60a/b) and one *pk2* allele (ANK40a/b) were each found in two identical copies. These results were confirmed by Southern blotting (Additional file 1: Figure S1) and are consistent with the sequencing of PCR products (Table 1).

**Table 1:** **List of*pk1*and*pk2*alleles associated to their prophages in different*Wolbachia*genomes**

***Wolbachia* strain**	**Super group**	**Host species**	**Genome**	**Prophage**	***pk1***		***pk2***		**Reference**
*w*CauB	B	*Cadra cautella*	ABA78515	WOCauB2	B2gp13		-		[26]
			ABA78516	WOCauB3	B2gp14		-		[26]
*w*Mel	A	*Drosophila melanogaster*	NC_002978	WOMelB	WD0596		WD0636		[11]
*w*Pip-BfB	B	*Culex pipiens pipiens*	-	-	AM397076	pk1b	AM397073	pk2c	[24]
*w*Pip-EpA	B	*Culex pipiens quinquefasciatus*	-	-	AM397075	pk1a	AM397074	pk2d	[24]
*w*Pip-Is	B	*Culex pipiens pipiens*	-	-	AM397078	pk1d	DQ000474	pk2a	[25]
*w*Pip-KaC	B	*Culex pipiens quinquefasciatus*	-	-	AM397079	pk1e	DQ000472	pk2a	[24]
*w*Pip-Lv	B	*Culex pipiens pipiens*	-	-	AM397077	pk1c	DQ000472	pk2a	[25]
*w*Pip-Pel	B	*Culex pipiens quinquefasciatus*	NC_010981	WOPip1	WPa_0256	ANK08	-		[8]
				WOPip2	WPa_0315	ANK14	WPa_0299	ANK12	[8]
				WOPip3	-		WPa_0340	ANK16	[8]
				WOPip4	-		WPa_0413	ANK25	[8]
				WOPip5	WPa_1308	ANK56	-		[8]
*w*Ri	A	*Drosophila simulans*	NC_012416	WORiA1	WRi_005620	ANK16	WRi_005440	ANK14	[9]
				WORiA2	WRi_010280	ANK35	WRi_010100	ANK33	[9]
				WORiB	WRi_p07220	ANK29	-		[9]
				WORiB	WRi_p07650	ANK31	-		[9]
*w*Pip-Sl	B	*Culex pipiens quinquefasciatus*	-	-	AM397076	pk1b	DQ000471	pk2b	[25]
*w*VitA	A	*Nasonia vitripennis*	HQ906662	WOVitA1		pk1-1		pk2-1	[27]
			HQ906663	WOVitA2		pk1-2		-	[27]
*w*VulC	B	*Armadillidium vulgare*	-	WOVulC1	**HM452371**	ANK46b	-		**This study**
				WOVulC2	**HM452372**	ANK32	-		**This study**
				WOVulC3	**HM452373**	ANK60b	**HM452376**	ANK40a	**This study**
				WOVulC4	**HM452374**	ANK25	**HM452376**	ANK40b	**This study**
				WOVulC5	**HM452371**	ANK46a	**HM452375**	ANK48	**This study**
				WOVulC6	**HM452373**	ANK60a	-		**This study**

Accession numbers from this study are in bold.

**Figure 1:**
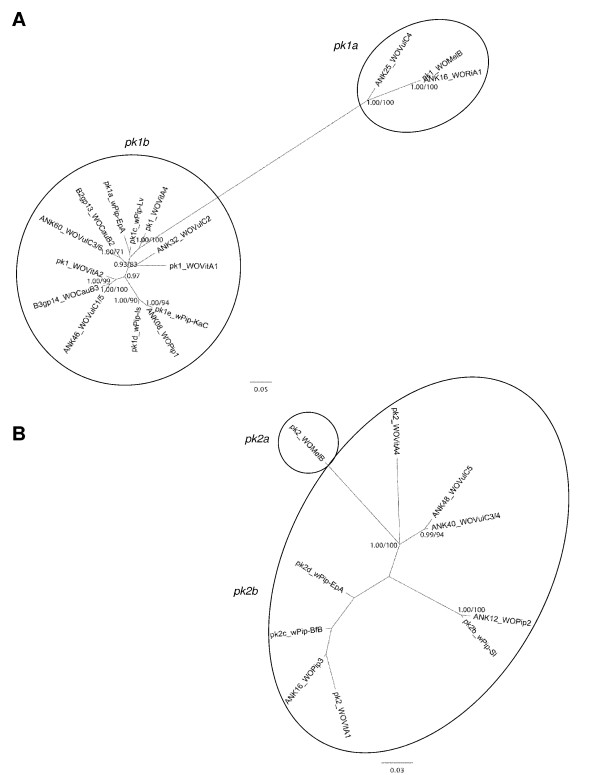
**Networks of*pk1*and*pk2*genes*.*** Networks of (**A**) pk1 and (**B**) pk2 sequences encoding ANK motif clusters. Identical sequences were merged and are represented under one unique label comprising information on the gene and prophage when available: (**A**) ANK16_WORiA1 corresponds also to ANK35_WORiA2, ANK08_WOPip1 = ANK14_WOPip2 = ANK56_WOPip5 = pk1b_wPip-BfB/Sl (B) pk2_WOMelB = ANK14_WORiA1 = ANK33_WORiA2, ANK12_WOPip2 = ANK25_WOPip4 = pk2_wPip-Is/Lv/KaC (see Table 1). Node support: ML bootstrap/MrBayes posterior probability, values <70 or 0.70 are not shown.

Relationships between these alleles and all the published sequences of *Wolbachia pk1* and *pk2* genes were assessed based on the DNA sequences encoding ANK-repeats. No evidence of recombination events has been detected in the alignments of *pk1* and *pk2* gene regions encoding ANK motifs (see Methods). In the *pk1* sequences, the number of variable sites is 467 out of 1068, of which 408 are informative. Similarly, there are 66 informative sites in the 292 bp-long *pk2* sequence alignment. The resulting genetic networks show that *pk1* and *pk2* sequences group in two clusters (Figure 1). Based on these relationships, we defined two different types of *pk1* genes (named *pk1a* and *pk1b*) and two different types of *pk2* genes (named *pk2a* and *pk2b*) (Figure 1). The *w*VulC genome contains a single copy of the *pk1a* type (ANK25) and 5 copies belonging to the *pk1b* type: ANK32, ANK46a/b and ANK60a/b (Figure 1A). The *w*VulC *pk1a* gene clusters together with the *pk1a* type gene of *w*Mel (Figure 1A, 15.9% divergent) whereas it shares 55.2 to 65.2 % identity with the five *w*VulC *pk1b* type sequences. The region encoding ANK repeats in the *w*VulC ANK60a/b alleles is related to the *pk1* sequence from the *w*CauB2 prophage (Figure 1A). *w*VulC ANK46a/b alleles are closely related to the *pk1* gene from the *w*CauB3 prophage (Figure 1A). ANK32 seems somehow related to the *pk1* gene from the WOVitA1 prophage (Figure 1A). The *w*VulC genome also harbours three genes of the *pk2b* type, ANK48 and ANK40a/b, further called *pk2b1* and *pk2b2* alleles respectively. In contrast to the *pk1* gene, all three *w*VulC *pk2* alleles form a cluster in the gene network (Figure 1B). Their closest relative is the *pk2* gene harboured by the WOVitA4 prophage of the *Wolbachia* strain endosymbiont of *Nasonia vitripennis* (Figure 1B).

Using the same primer set as for *w*VulC ( Additional file 1: Table S1), the taxonomic distribution of *pk1* and *pk2* genes was extended by PCR to seven *Wolbachia* strains that induce either CI or feminization in isopods. All these strains of isopods are known to belong to the B-supergroup of *Wolbachia* whatever the phylogenetic marker used [2]. They do not form separate monophyletic clades according to the phenotype they induce in their hosts based on the *wsp* gene ( Additional file 1: Figure S2). We also investigated the copy number variation by Southern blot analyses of *Eco*RI or *Bam*HI digested DNA using *pk1apk1b* and *pk2b1* probes which, according to sequence identities, preferentially hybridized on *pk1apk1b* and *pk2b* types, respectively (Table 2 & Additional file 1: Figure S1). In congruence with amplification and sequencing data, the *pk1a* and *pk1b* probes revealed two to six copies of the *pk1* gene in the studied strains (Table 2). By direct sequencing of the PCR products, we found that the *pk1a* gene of *Wolbachia* strains of *C. convexusP. pruinosusA. vulgare* (*w*VulM) and *A. nasatum* harboured 1, 1, 2 and 3 *Eco*RI sites, respectively, explaining the discrepancy between the number of bands observed by Southern blots, and the number of different sequences obtained (Table 2 & Additional file 1: Figure S1). Similarly, two *pk1b* alleles of the *Wolbachia* strain of *A. nasatum* contained one *Bam*HI restriction site. Each of the two more intense Southern Blot signals ( Additional file 1: Figure S1) revealed the presence of two identical copies *w*VulC *pk1b* alleles, as confirmed by the analysis of contigs. Furthermore, Southern blots using a *pk2b1* probe in combination with sequencing data revealed three copies of the *pk2* gene in all strains tested except one (Table 2 & Additional file 1: Figure S1). In the *Wolbachia* strain of *P. pruinosus*, sequences of PCR products revealed two identical *pk2* alleles, each containing one *Bam*HI restriction site explaining the five signals obtained by Southern blotting (Table 2 & Additional file 1: Figure S1). Moreover, no signal was obtained from digested and undigested DNA of *Wolbachia*-free ovaries of isopod (non-infected population from Nice, France), which confirmed the *Wolbachia* origin of the *pk1 and pk2* genes.

**Table 2:** Copy number of *pk1* and *pk2* alleles according to their ‘type’ in different *Wolbachia* strains

***Wolbachia* strain**	**Host**	***pk1***	**alleles**	***pk2***	**alleles**
		**a**	**b**	**a**	**b**
*w*Ase	*Oniscus asellus*	1	3	0	3
*w*CauB	*Cadra cautella*	0	2	0	0
*w*ConV	*Cylisticus convexus*	1	3	0	3
*w*Dil	*Porcellio dilatatus dilatatus*	1	4	0	3
*w*Mel	*Drosophila melanogaster*	1	0	1	0
*w*Nas	*Armadillidium nasatum*	1	5	0	3
*w*Pet	*Porcellio dilatatus petiti*	1	1	0	3
*w*Pip-Pel	*Culex pipiens*	0	3	0	3
*w*Prull	*Porcellionides pruinosus*	1	5	0	3
*w*Ri	*Drosophila simulans*	3(2)	1	2	0
*w*VitA	*Nasonia vitripennis*	3	0	2	0
*w*VulC	*Armadillidium vulgare*	1	5	0	3
*w*VulM	*Armadillidium vulgare*	1	5	0	3

The number of pseudogenes is indicated in brackets. ND: non determined.

### Molecular evolution of *pk1* and *pk2*genes

The GC content of *w*VulC *pk1* alleles (mean ± SE, 33.9 ± 0.3%) is similar to that of the whole genome assembly (34.5%) whereas the GC content of *w*VulC *pk2* alleles (ANK40a/b: 36.8%, ANK48: 36.3%) is significantly greater. Similar results were obtained considering *pk1* and *pk2* genes of all *Wolbachia* genomes (*pk1*: 34.0 ± 0.1%; *pk2*: 37.2 ± 0.2%; genomes: 34.8 ± 0.3%) (paired t-test, t = 13.79, df = 15, p = 6.3e^-10^) ( Additional file 1: Table S2). Interestingly, the GC content of *pk1* and *pk2* sequences is significantly different from the whole prophage sequences, which comprise an intermediate GC content of 35.8 ± 0.2% (paired t-tests; prophage *vs. pk1*, t = 12.60, df = 11, p = 7.0e^-8^; prophage *vs. pk2*, t = 3.85, df = 8, p = 4.9e^-3^) ( Additional file 1: Table S2). ANK motif-encoding sequence analysis indicated no recombination and Ka/Ks (the ratio of the rate of non-synonymous substitutions (Ka) to the rate of synonymous substitutions (Ks)) of all positions was 0.211 ± 0.009 for Pk1 and 0.245 ± 0.020 for Pk2. Purifying selection is thus acting on these domain-encoding sequences and no sites are under positive selection.

All translated *pk1* full-length sequences are predicted to harbour two transmembrane domains in their C-terminal region but a variable number of ANK motifs ranging from 8 to 10 ( Additional file 1: Figure S3). In *w*VulC, ANK46a/b and ANK60a/b sequences (*pk1b* type) are shorter in their N-terminal region than the other Pk1 translated sequences (42 and 62 amino acids, respectively). One indel at position 117 of the DNA sequence of *w*VulC ANK46a/b is responsible for a frame shift, which splits the gene into two ORFs homologous to the full-length *pk1* of other strains. ANK60a/b sequences are shortened by a transposase gene insertion in the 5′ region. In contrast, *pk2* translated sequences are more conserved (84.5 to 100% identity) among *Wolbachia* strains than *pk1*. All Pk2 amino acid sequences harbour 3 ANK motifs except in the *w*Au strain (host: *D. simulans*) in which a premature stop codon disrupts the third motif ( Additional file 1: Figure S3).

### Comparative analysis of *pk1* and *pk2* mRNA expression in CI and feminizing *Wolbachia* strains

**Figure 2:**
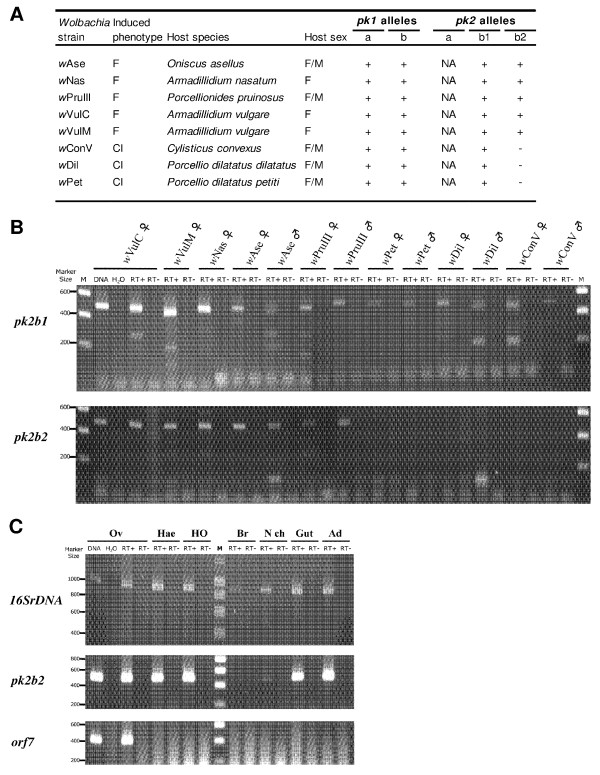
**Transcriptional analyses of*pk1*and*pk2*alleles.** (**A**) Transcriptional results of the *pk1* and *pk2* alleles obtained from gonads of eight isopod species harbouring either feminizing (F) or CI-inducing (CI) *Wolbachia* strains. Plus or minus signals indicate expression, or not, of the copy(ies). Distinction is made between the two different *pk2* alleles named *pk2*b1 and *pk2*b2 within the pk2b type. F: female; M: male. NA: no pk2a type alleles were amplified in these strains. (**B**) Transcriptional results of *pk2b1* and *pk2b2* alleles are shown from ovaries or testes (when infected) of eight isopod species. Primers used are shown in ( Additional file 1: Table S1). The DNA template control (only *w*VulC presented) shows the intensity and specificity of the band detected with each pair of primers. RT + and RT- indicate, respectively, the presence or the absence of reverse transcriptase in the reactions. M: DNA size markers. (**C**) Transcriptional results of the *16S* rDNA, *pk2b2* and *orf7* genes in seven different tissues of *A. vulgare* harbouring the *w*VulC *Wolbachia* strain. Ov: ovaries; Hae: haemocytes; HO: hematopoietic organ; Br: brain; N ch: nerve chain; gut; Ad: adipose tissue.

RT-PCR using allele-specific primers was performed to examine the expression patterns of *pk1* and *pk2* mRNA in adult gonads of isopods harbouring CI-inducing or feminizing *Wolbachia* strains (Figure 2). Evidence of expression was observed for all copies of *pk1* and *pk2* genes except for one allele of the *pk2b* type (Figure 2A). Although the *pk2* alleles were amplified by PCR using DNA of all the three isopod CI-inducing *Wolbachia* strains (positive controls only shown for *w*VulC), RT-PCR did not yield any detectable products in these strains using the *pk2b2* allele-specific primers (Figure 2B). In contrast, the *pk2b2* allele was clearly expressed in all the feminizing *Wolbachia* strains (Figure 2B). In hosts where both males and females are infected by CI-inducing or feminizing strains, no clear sex-specific differences were observed in *pk1* and *pk2* expression (Figure 2A). We further examined the expression of *pk2b2* and another prophage gene, *orf7* which encodes the phage capsid, in several tissues of *A. vulgare* females harbouring the feminizing *w*VulC strain (Figure 2C). While *orf7* was expressed only in ovaries, the host tissue where the density of *Wolbachia* is higher, transcription of *pk2b2* was revealed in all tissues tested (except the brain) (Figure 2C).

## Discussion

In this study, we found that a large copy number variation of *pk1* and *pk2* genes exists among *Wolbachia* strains, which is probably coupled to prophage dynamics and evolution. Copy number divergence in the ankyrin *pk1* and *pk2* is consistent with the results of previous Southern blotting analyses using the minor capsid *orf7* phage gene [28]. Four different *orf7* paralogs had already been identified in the *w*VulC strain through cloning and sequencing of heterogeneous PCR products [28]. Since multiple infections of *Wolbachia* in a single individual have never been observed in isopods, we can conclude that the phage WO is likely to be present in several copies in each *Wolbachia* strain. Our observations of *Wolbachia* strains of isopods suggest that dynamics of the prophage *pk1* and *pk2* genes is similar to that observed in the *w*Ri and *w*Pip-Pel genomes [8,9]. While the *pk1* and *pk2 ank* genes are each found as a single copy within the WO-B prophage of the *w*Mel genome [11], sequencing of the CI-inducing *w*Pip-Pel, *w*Ri and *w*VitA *Wolbachia* prophages revealed a duplication of these genes together with the WO-B-like prophages [8,9,27]. For instance, in the *w*Pip-Pel genome, the three *pk1* and the three *pk2* genes are spread among the five different prophages which are closely related to the WO-B *w*Mel prophage [8]. Hence, the divergence in the *pk1* and *pk2* gene copy number between *Wolbachia* strains may be explained by mechanisms related to bacterial genome organization and modulation of gene copy number [26,29-32]. As an example, two pseudogenes (wRi_ANK29 and ANK31) out the four copies of the *pk1* gene in *w*Ri, are spread in the WORiB prophage (previously annotated WO-C prophage [9], see Table 1) and may have originally been a single *pk1* gene further disrupted by an insertion sequence ISWpi7. On the other hand, the high GC content of *pk2* supports the occurrence of recent lateral transfers of prophage fragments containing the *pk2* gene but not necessarily *pk1* in the *Wolbachia* genomes. However, we cannot exclude the hypothesis that linkage disequilibrium occurs between *pk1* and *pk2* genes that are separated by at least 6.7 kilobases, representing less than 0.04% of the whole genome size. These results also highlight the genomic plasticity of the prophage region among *Wolbachia* strains as part of the global plasticity observed in the *Wolbachia* genomes [33]. Maintenance of such “mobile elements” in *Wolbachia* strains of arthropods may be due to the absence of, or a reduced efficiency of selection on the prophages. Nevertheless, the purifying selection acting on these *pk1* and *pk2* genes suggest that maintenance of sequences confers an adaptive advantage.

Besides identifying mosaic prophages, our results also reveal the differential expression of one *pk2* ankyrin according to the *Wolbachia* phenotype they induce (CI *vs.* feminization). One allele (*pk2b2*) is only expressed in the feminizing strains and never in the three CI-inducing strains of isopods. In contrast to the observations for *w*Pip [22,23], expression pattern of *pk2b2* suggests that this allele is not involved in CI in isopods. In two recent studies, it has been shown that expression of *pk1* and *pk2* genes from *w*Mel was not correlated with the CI phenotype in *D. melanogaster*[34,35]. Our transcriptional result rather leads to the hypothesis that this *pk2b2* allele is involved in the feminization of isopod hosts. This hypothesis is strengthened by the observation that the *pk2b2* allele is expressed in all *A. vulgare* tissues (except in the brain) whereas another prophage gene (*orf7*) is only expressed in ovaries. Furthermore, no differential expression of *pk1* and *pk2* genes was identified between sexes in isopods when either CI-inducing or feminizing *Wolbachia* infects both males and females. This result differs from those of Sinkins and colleagues who showed that in some CI-inducing *w*Pip variants, the three *pk2* genes (the two identical *w*Pip_ANK12 and *w*Pip_ANK25, and *w*Pip_ANK16) are highly expressed in females but never in males [22,23].

Our data do not enable us to explain why *pk2b2* is only expressed in feminizing strains of *Wolbachia* whereas its homologs, also found in CI-inducing strains, are associated with CI phenotype in mosquitoes [22,23]. First, one can suggest that this allele has been inactivated or importantly down regulated in the CI-inducing strains of isopods. Change in regulatory element repertoire and divergence in patterns of expression may occur after small-scale duplication of the genome [36]. A corollary to a change in location, paralogous and homologous *pk2* copies within and among *Wolbachia* strains would have followed different evolutionary trajectories leading to such a phenotypic diversity. Second, genomic imprinting, process by which genes are expressed from only one parental allele due to epigenetic mechanism, can be considered as a molecular mechanism underlying the diversity of phenotypes. Recently, early changes in gene imprinting and aberrant expression of specific genes have been shown to be coupled to parthenogenesis in mice embryos [37]. Third, one can suggest that genes in the *pk2* family could have diverse functions. In this way, post-transcriptional modifications and dosage of *Wolbachia* products, as well as genetic control by the host, cannot be dismissed. As previously suggested [38], differences in *Wolbachia*-induced feminization as well as the presence of the bacteria in *O. asellus* males, may simply result from differences in bacterial dosage or in host targets. The basic molecular mechanisms that mediate *Wolbachia* feminization are also still unknown although it is unlikely that this effect is driven by only one gene. In *A. vulgareWolbachia* effectors may target the proteinaceous androgenic hormone or its receptor, or another major sex determinant, thereby inhibiting the androgenic gland differentiation and preventing the androgenic hormone from reaching the target tissues such as gonads and tegumental epithelium [2,39,40]. This hypothesis suggests a late action of feminizing *Wolbachia* on host target(s) during its development, as opposed to the very early action of other *Wolbachia* strains that induce parthenogenesis, CI or male killing [5,41].

## Conclusions

Our results highlight a large copy number variation of both *pk1* and *pk2* genes among strains, likely linked to prophage diversity, and also the specific expression of one *pk2* allele only in the feminizing *Wolbachia* strains of isopods. This correlation supports the hypothesis that phenotype-related effectors or specific strain determinants in *Wolbachia* are likely to be encoded by prophage genes, ankyrin-repeat encoding genes, and predicted genes of unknown function [42]. Our results thus reveal the need to search for host molecules targeted by *Wolbachia* ankyrins and their functions with respect to host sex manipulation by *Wolbachia*.

## Methods

### *Wolbachia*-infected isopod species

All isopods used in this study were collected in France and reared in the laboratory. To date, CI *Wolbachia* have been described in three species: *Cylisticus convexus* (*w*ConV, Villedaigne) [41], *Porcellio dilatatus petiti* (*w*Pet, Saint-Honorat) [43] and *P. d. dilatatus* (*w*Dil, Sainte-Marguerite) (Grève, unpublished results). *Wolbachia* strains inducing feminization have been described in *A. vulgare* (*w*VulC, Celles sur Belle and *w*VulM, Mery sur Cher) [44,45], *A. nasatum* (*w*Nas, Poitiers) [46], *Oniscus asellus* (*w*Ase, Quinçay) [38], *Porcellionides pruinosus* (*w*PruIII, Nevers) [47]. An uninfected lineage of *A. vulgare* (originating from Nice, France) was used as negative control for PCR and Southern blotting experiments.

Total DNA was extracted from male and female gonads of all isopod species as described previously [48]. Infection status of each individual was confirmed by a PCR-assay based on the bacterial 16S rDNA gene using *Wolbachia*-specific primers ( Additional file 1: Table S1) [49].

### Distribution of *pk1* and *pk2* genes

The genome of the feminizing *w*VulC *Wolbachia* strain is at the final assembly step (whole-genome shotgun-sequencing project: European *Wolbachia* EuWol (contract QLK3-CT2000-01079, coordinated by K. Bourtzis, University of Ioannina, Greece). This includes phage contigs of which sequences are homologous to the *Wolbachia* WO prophage. Annotation of the *pk1* and *pk2* genes was performed by protein and DNA homology searches with BLASTP and BLASTN programs [50] using the *w*Pip-Pel *pk1* and *pk2* alleles as queries (see Table 1). Ankyrin and other functional motif predictions were performed by the SMART web server [51] on protein sequences.

Specific primers were designed to amplify full-length or 200–500 bp fragments of the *w*VulC *pk1* and *pk2* alleles using a standard PCR protocol as previously described ( Additional file 1: Table S1) [52]. The purified PCR products were directly sequenced on both strands on an ABI PRISM 3100 Genetic Analyzer using Big Dye Terminator v3.1 Cycle Sequencing Kit (Applied Biosystems) according to the manufacturer’s instructions.

*pk1* or *pk2* copy number variation among *Wolbachia* strains was assessed by Southern blotting. About 15 μg of total DNA were digested at 37° overnight with *Eco*RI or *Bam*HI enzymes that did not cut any of the *w*VulC *pk1* and *pk2* alleles. Digested DNA as well as undigested DNA from non-infected ovaries used as controls (data not shown) was electrophoresed on 0.8% agarose gels and blotted to nylon membranes. Probes were obtained by PCR amplification of the *w*VulC full-length *pk1* (*pk1a* and *pk1b* types) and *pk2* (*pk2b* type) *ank* genes ( Additional file 1: Table S1), labelled using [α-^32^P]-dCTP by the random primer method and hybridized overnight to membranes. The final wash was performed at 52° in 0.1X SSC. Hybridized blots were imaged and analyzed using a PhosphoImager (Molecular Dynamics, Sunnyval, CA, USA).

### Sequence analyses of *pk1* and *pk2* genes

Homologous sequences of both genes were first aligned in the server-based program MAFFT (http://align.bmr.kyushu-u.ac.jp/mafft/online/server/) using automatic settings. The resulting matrices were manually checked according to the predicted amino acid translation using BioEdit v7.0.9 [53]. MEGA5 software [54] was used to calculate nucleotide sequence divergence. For each locus, the GC content, the number of variable sites and the level of nucleotide diversity per site (Pi) were calculated. Ka/Ks likelihood analysis was also performed using the Selecton web server [55].

Recombination analysis was performed with RDP version v3.42 [56] using an alignment of non-redundant *pk1* and *pk2* nucleotide region encoding ANK-repeat domains. The parameters were set as follows: sequences were considered linear, the highest acceptable P value cut-off was 0.01, a Bonferroni correction was applied, consensus daughter sequences were found, gaps were included, different window sizes of variable sites were tested and 1,000 permutations were performed.

The best-fitted model of DNA evolution was estimated with jModelTest v0.1.1 [57] according to the corrected Akaike Information Criterion [58]. The selected model was TIM + G for *pk1* and HKY + I for the *pk2* locus encoding the ANK domain cluster. Gene genealogies were constructed using MrBayes v3.1.2 software [59,60] and supported by Bayesian and Maximum likelihood (ML) probabilities. Two Metropolis-coupled Markov chain Monte Carlo (MCMC) analyses were run for 5,000,000 generations and sampled every 250 generations. The first 25% of sampled trees were considered burn-in trees and were discarded before constructing a 50% majority rule consensus tree. ML analyses were carried out in PhyML 3.0 [61]. Node support came from 1,000 multiparametric bootstrap replicates. The networks were visualized with FigTree v1.3.1 (http://tree.bio.ed.ac.uk/software/figtree). The network tree of the *wsp* gene was built following an identical Bayesian methodology (model: TPM3uf + I + G) ( Additional file 1: Figure S2).

### Expression of ankyrin genes

Total RNAs were isolated from 20 to 50 gonads dissected from all species using the RNeasy Mini Kit (Qiagen) according to the manufacturer’s instructions. Ovaries were used in *A. vulgare* and *A. nasatum* where only females are infected. After a treatment with DNaseI (2U/μL, Ambion) at 37° for 30 min, 1 μg of RNA was used for reverse transcription using Superscript III kit (Invitrogen) as described by the manufacturer. To determine the expression of each gene, 1 μL of the reverse transcriptase reaction was used as template for the RT-PCR experiments. Control of the RT reactions was performed by omitting reverse transcriptase in the negative (RT-) controls and by testing the expression of the *Wolbachia* 16S rDNA gene ( Additional file 1: Table S1). Genomic DNA of all species was also used as a positive control of the PCR reactions as well as the one of the uninfected population (Nice, France) as negative control. Transcriptional analyses of *pk2b2* and *orf7* genes in several tissues of *A. vulgare* harbouring the feminizing *w*VulC *Wolbachia* strain were run as previously described [52]. The *16S* rDNA gene expression was again used to check the quality of the extracted RNA in all these tissues.

## Authors’ contributions

SP carried out the molecular genetic studies, participated in the data acquisition and performed all analyses and drafted the manuscript. CL and LC participated in the data acquisition. RAG was involved in project conception and critical revision of the manuscript. PG and DB coordinated the study, participated in its design, in the data acquisition and drafted the manuscript. All authors read and approved the final manuscript.

## Supplementary Material

Additional file 1 **Figure S1. Southern blotting analyses*.*** Reconstituted Southern blots of *Eco*RI or *Bam*HI digested DNA from 8 *Wolbachia*-infected terrestrial isopod species hybridized with three different probes (see text for details). White triangles highlight positions of the hybridized fragments. Lanes were loaded with DNA from *Wolbachia* strain endosymbionts of PDP as *P. dilatatus petiti*; PDD as *P. dilatatus dilatatus*; CC as *C. convexus*; AVC as *A. vulgare* strain *w*VulC; AVM as *A. vulgare* strain *w*VulM; AN as *A. nasatum*; OA as *O. asellus*; PP as *P. pruinosus* strain *w*PruIII. The number of bands in some lanes is higher than the number of copies presented in Table 2 due to *Eco*RI and/or *Bam*HI restriction site(s) in these copies, as confirmed by sequencing. Upper light bands correspond to partially digested DNA fragments. **Figure S2. Phylogenetic tree of*Wolbachia*strains based on the*wsp*gene.***Wolbachia* strains of isopods are shown in bold (*w*Album: *Armadillidium album*; *w*Ase: *Oniscus asellus*; *w*ConV: *Cylisticus convexus*; *w*Dil: *Porcellio dilatatus dilatatus*; *w*Elo: *Chaetophiloscia elongata*; *w*Hoo: *Sphaeroma hookeri*; *w*Mus: *Philoscia muscorum*; *w*Nas: *Armadillidium nasatum*; *w*Oce: *Ligia oceanica*; *w*Pet: *Porcellio dilatatus petiti*; *w*PruIII: *Porcellionides pruinosus*; *w*Rug: *Sphaeroma rugicauda*; *w*Scaber: *Porcellio scaber*; *w*VulC, *w*VulM, *w*VulP: *Armadillidium vulgare*). The additional B-supergroup *Wolbachia* strains and the host phenotypes they induce are based on previously published information (*w*AlbB: *Aedes albopictus*; *w*Alt: *Chelymorpha alternans*; *w*Au, *w*Ma, *w*No, *w*Ri: *Drosophila simulans*; *w*Bol: *Hypolimnas bolina*; *w*CauB: *Cadra cautella*; *w*Con: *Tribolium confusum*; *w*Dei: *Trichogramma deion*; *w*Enc: *Acraea encedon*; *w*For: *Encarsia formosa*; *w*Fir: *Gryllus firmus*; *w*Kue: *Ephestia kuehniella*; *w*Mel: *Drosophila melanogaster*; *w*Ori: *Tagosodes orizicolus*; *w*Pip-JHB, *w*Pip-Pel: *Culex pipiens quinquefasciatus*; *w*Scap: *Ostrinia scapulalis*; *w*Sn: *Drosophila sechellia*; *w*Stri: *Laodelphax striatellus*; *w*Tai: *Teleogryllus taiwanemma*; *w*VitA: *Nasonia vitripennis*). Confirmed or suspected induced-phenotypes of *Wolbachia* strains of isopods are drawn from Bouchon *et al.* (2008). The red colour of strains corresponds to the feminizing induced-phenotype, blue to CI, green to male killing, light grey to parthenogenesis, and black to suspected feminization. Node supports are shown by posterior probabilities from Bayesian inferences. **Figure S3. SMART outputs representing the number of ANK motifs found in Pk1 translated sequences**. **Figure S4. SMART outputs representing the number of ANK motifs found in Pk2 translated sequences**. **Table S1. List of primers used in this study for sequencing (PCR), for expression analyses (RT-PCR), or for Southern blots (SB).** Expected PCR product size in base pair (bp) was calculated relative to the *w*VulC reference sequences. **Table S2. List of*pk1*and*pk2*sequences used for Figure 1,** Additional file 1**: Figure S3 and** Additional file 1**: Figure S4.** Accession numbers from this study are in bold. Click here for file
