# Supplementary material for: The expression of one ankyrin pk2 allele of the WO prophage is correlated with the Wolbachia feminizing effect in isopods
Source: BMC Microbiol. 2012 Apr 12;12:55. doi: 10.1186/1471-2180-12-55 (PMC3431249; doi:10.1186/1471-2180-12-55)
Supplement: Additional file 1 — Figure S1. Southern blotting analyses. Reconstituted Southern blots of EcoRI or BamHI digested DNA from 8 Wolbachia-infected terrestrial isopod species hybridized with three different probes (see text for details). White triangles highlight positions of the hybridized fragments. Lanes were loaded with DNA from Wolbachia strain endosymbionts of PDP as P. dilatatus petiti; PDD as P. dilatatus dilatatus; CC as C. convexus; AVC as A. vulgare strain wVulC; AVM as A. vulgare strain wVulM; AN as A. nasatum; OA as O. asellus; PP as P. pruinosus strain wPruIII. The number of bands in some lanes is higher than the number of copies presented in Table 2 due to EcoRI and/or BamHI restriction site(s) in these copies, as confirmed by sequencing. Upper light bands correspond to partially digested DNA fragments. Figure S2. Phylogenetic tree ofWolbachiastrains based on thewspgene.Wolbachia strains of isopods are shown in bold (wAlbum: Armadillidium album; wAse: Oniscus asellus; wConV: Cylisticus convexus; wDil: Porcellio dilatatus dilatatus; wElo: Chaetophiloscia elongata; wHoo: Sphaeroma hookeri; wMus: Philoscia muscorum; wNas: Armadillidium nasatum; wOce: Ligia oceanica; wPet: Porcellio dilatatus petiti; wPruIII: Porcellionides pruinosus; wRug: Sphaeroma rugicauda; wScaber: Porcellio scaber; wVulC, wVulM, wVulP: Armadillidium vulgare). The additional B-supergroup Wolbachia strains and the host phenotypes they induce are based on previously published information (wAlbB: Aedes albopictus; wAlt: Chelymorpha alternans; wAu, wMa, wNo, wRi: Drosophila simulans; wBol: Hypolimnas bolina; wCauB: Cadra cautella; wCon: Tribolium confusum; wDei: Trichogramma deion; wEnc: Acraea encedon; wFor: Encarsia formosa; wFir: Gryllus firmus; wKue: Ephestia kuehniella; wMel: Drosophila melanogaster; wOri: Tagosodes orizicolus; wPip-JHB, wPip-Pel: Culex pipiens quinquefasciatus; wScap: Ostrinia scapulalis; wSn: Drosophila sechellia; wStri: Laodelphax striatellus; wTai: Teleogryllus taiwanemma; wVitA: Naso [file 1471-2180-12-55-S1.doc]

Figure S1.—Southern blotting analyses.


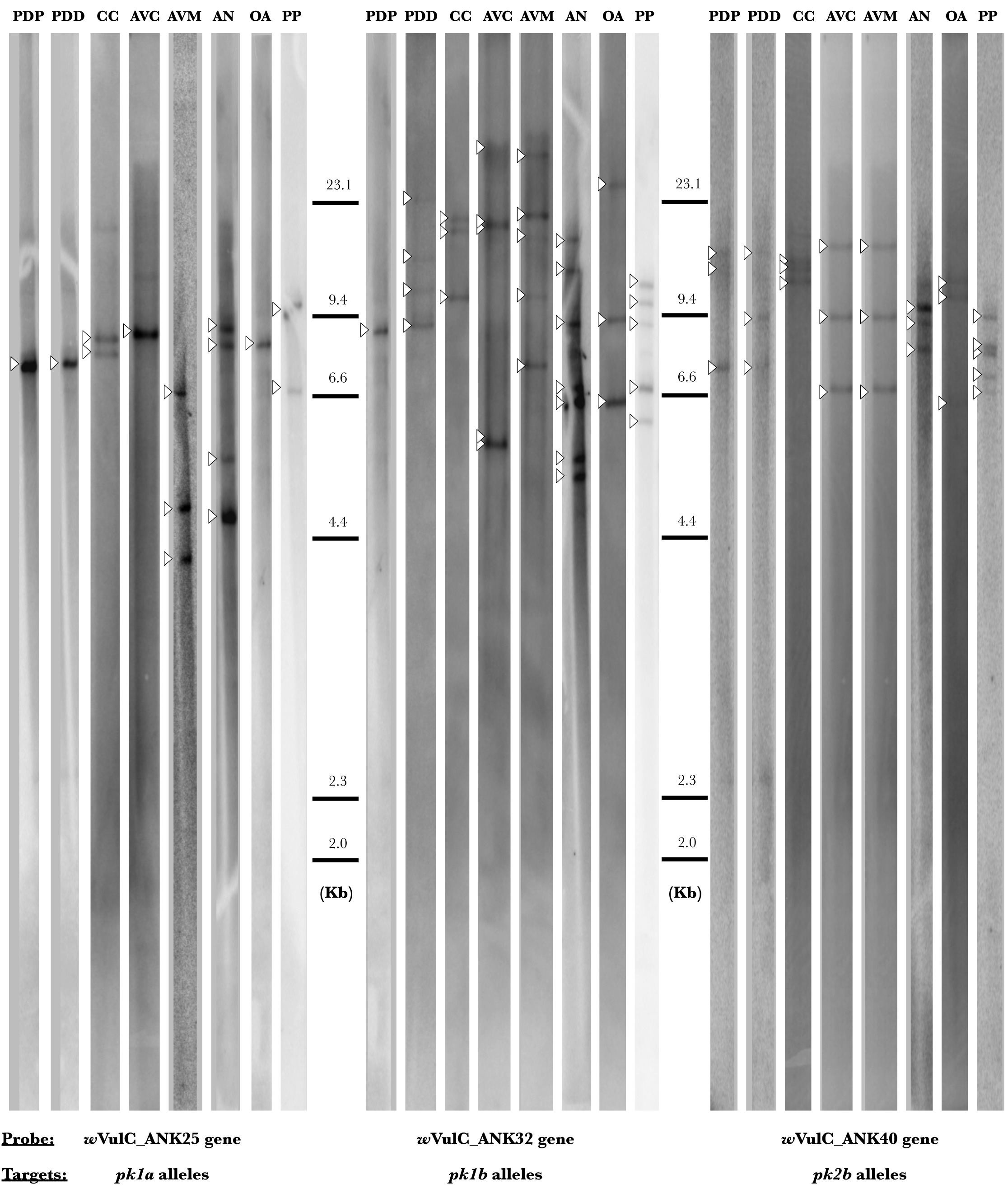


Reconstituted Southern blots of *Eco*RI or *Bam*HI digested DNA from 8 *Wolbachia*-infected terrestrial isopod species hybridized with three different probes (see text for details). White triangles highlight positions of the hybridized fragments. Lanes were loaded with DNA from *Wolbachia* strain endosymbionts of PDP as *P. dilatatus petiti*; PDD as *P. dilatatus dilatatus*; CC as *C. convexus*; AVC as *A. vulgare* strain *w*VulC; AVM as *A. vulgare* strain *w*VulM; AN as *A. nasatum*; OA as *O. asellus*; PP as *P. pruinosus* strain *w*PruIII. The number of bands in some lanes is higher than the number of copies presented in Table 2 due to *Eco*RI and/or *Bam*HI restriction site(s) in these copies, as confirmed by sequencing. Upper light bands correspond to partially digested DNA fragments.

Figure S2. —Phylogenetic tree of *Wolbachia* strains based on the *wsp* gene.


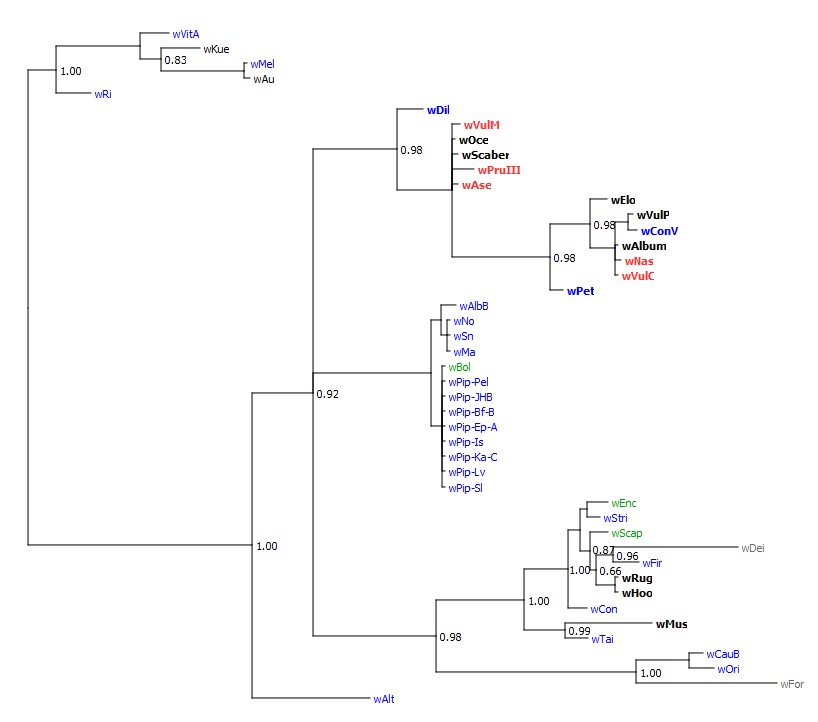


Phylogenetic tree of *wsp* sequences identified by their *Wolbachia* strain names. *Wolbachia* strains of isopods are shown in bold (*w*Album: *Armadillidium album*; *w*Ase: *Oniscus asellus*; *w*ConV: *Cylisticus convexus*; *w*Dil: *Porcellio dilatatus dilatatus*; *w*Elo: *Chaetophiloscia elongata*; *w*Hoo: *Sphaeroma hookeri*; *w*Mus: *Philoscia muscorum*; *w*Nas: *Armadillidium nasatum*; *w*Oce: *Ligia oceanica*; *w*Pet: *Porcellio dilatatus petiti*; *w*PruIII: *Porcellionides pruinosus*; *w*Rug: *Sphaeroma rugicauda*; *w*Scaber: *Porcellio scaber*; *w*VulC, *w*VulM, *w*VulP: *Armadillidium vulgare*). The additional B-supergroup *Wolbachia* strains and the host phenotypes they induce are based on previously published information (*w*AlbB: *Aedes albopictus*; *w*Alt: *Chelymorpha alternans*; *w*Au, *w*Ma, *w*No, *w*Ri: *Drosophila simulans*; *w*Bol: *Hypolimnas bolina*; *w*CauB: *Cadra cautella*; *w*Con: *Tribolium confusum*; *w*Dei: *Trichogramma deion*; *w*Enc: *Acraea encedon*; *w*For: *Encarsia formosa*; *w*Fir: *Gryllus firmus*; *w*Kue: *Ephestia kuehniella*; *w*Mel: *Drosophila melanogaster*; *w*Ori: *Tagosodes orizicolus*; *w*Pip-JHB, *w*Pip-Pel: *Culex pipiens quinquefasciatus*; *w*Scap: *Ostrinia scapulalis*; *w*Sn: *Drosophila sechellia*; *w*Stri: *Laodelphax striatellus*; *w*Tai: *Teleogryllus taiwanemma*; *w*VitA: *Nasonia vitripennis*). Confirmed or suspected induced-phenotypes of *Wolbachia* strains of isopods are drawn from Bouchon *et al.* (2008). The red colour of strains corresponds to the feminizing induced-phenotype, blue to CI, green to male killing, light grey to parthenogenesis, black to suspected feminization. Node supports are shown by posterior probabilities from Bayesian inferences.

Figure S3. —SMART outputs representing the number of ANK motifs found in Pk1 translated sequences.





Figure S4. —SMART outputs representing the number of ANK motifs found in Pk2 translated sequences.





Table S1. List of primers used in this study for sequencing (PCR), for expression analyses (RT-PCR), or for Southern blots (SB). Expected PCR product size in base pair (bp) was calculated relative to the *w*VulC reference sequences.

Table S2. List of *pk1* sequences used for Figure 1 and Figure S2. Redundant sequences are under a unique sequence name. Accession numbers from this study are in bold.
